# CCR-CCL axes as key upstream influencers of pancreatic ductal adenocarcinoma: CCR2–CCL2, CCR5–CCL5, CCR4–CCL17/22, CCR6–CCL20, CCR7–CCL19/21

**DOI:** 10.3389/fimmu.2026.1713123

**Published:** 2026-01-29

**Authors:** Ingyu Bahng

**Affiliations:** Department of Molecular and Cell Biology, University of California, Berkeley, Berkeley, CA, United States

**Keywords:** chemokine receptor antagonists, chemokine signaling, extracellular matrix (ECM), immune evasion, pancreatic ductal adenocarcinoma (PDAC), tumor-associated macrophages (TAMs)

## Abstract

PDAC remains one of the most lethal malignancies, characterized by a highly desmoplastic ECM that promotes an immunosuppressive signaling network and dampens the effectiveness of traditional therapies. Among the several key contributors to its immune evasion pathways are chemokine signaling axes, which orchestrate the recruitment of regulatory immune cell populations, promote metastasis, and remodel the TME in favor of tumor progression. This review comprehensively examines the roles of major CCR-CCL signaling pathways—primarily focusing on the CCR2–CCL2, CCR5–CCL5, CCR4–CCL17/22, CCR6–CCL20, and CCR7–CCL19/21 axes—in PDAC development, detailing their expression patterns, immunologic impact, and downstream signaling mechanisms and outcomes. We further detail past and ongoing therapeutic efforts and trials addressing these axes in both PDAC and relevant non-PDAC settings via several small-molecule antagonists and monoclonal antibodies: BMS-813160, Maraviroc, Leronlimab, FLX475, PF-07054894, IDOR- 1117-2520, and CAP-100. Despite continuous advances in the field, the current body of evidence remains limited and presents significant research gaps in areas such as spatial profiling, stage-specific analyses, and general mechanistic validation in PDAC-specific settings. Addressing these shortcomings will be key to developing a more comprehensive knowledge of the field and improving future therapeutic strategies to overcome PDAC.

## 1 Introduction

Pancreatic ductal adenocarcinoma (PDAC) is one of the most aggressive cancers worldwide, ranking sixth among the leading causes of cancer-related mortality (1). In 2022 alone, PDAC was responsible for over 510,000 new cases and roughly 467,000 deaths worldwide, with incidence rates projected to increase by 95.4% by 2050 (1). The overall five-year survival rate for all stages of pancreatic cancer in 2011 was 4.2%, and only marginal improvements have come to fruition from the advances made in chemotherapy and immunotherapy in recent years (2, 3). While early-stage diagnoses—stages IA and IB—have seen modest survival gains from 47% to 75% and from 38% to 68% between 2004 and 2015, respectively, over 80% of patients still present with advanced or metastatic disease, where median survival drops to a few months (2, 4). Surgical resection remains the only potentially curative intervention. Yet, only around 20% of patients are eligible for resection at diagnosis due to the extent of local invasion or remote metastasis (5, 6). This aggressive progression is tightly linked to the immunosuppressive PDAC tumor microenvironment (TME), characterized by dense desmoplasia, regulatory cell infiltration, and a signaling network that collectively suppresses anti-tumor responses (3, 7, 8).

**Figure 1:**
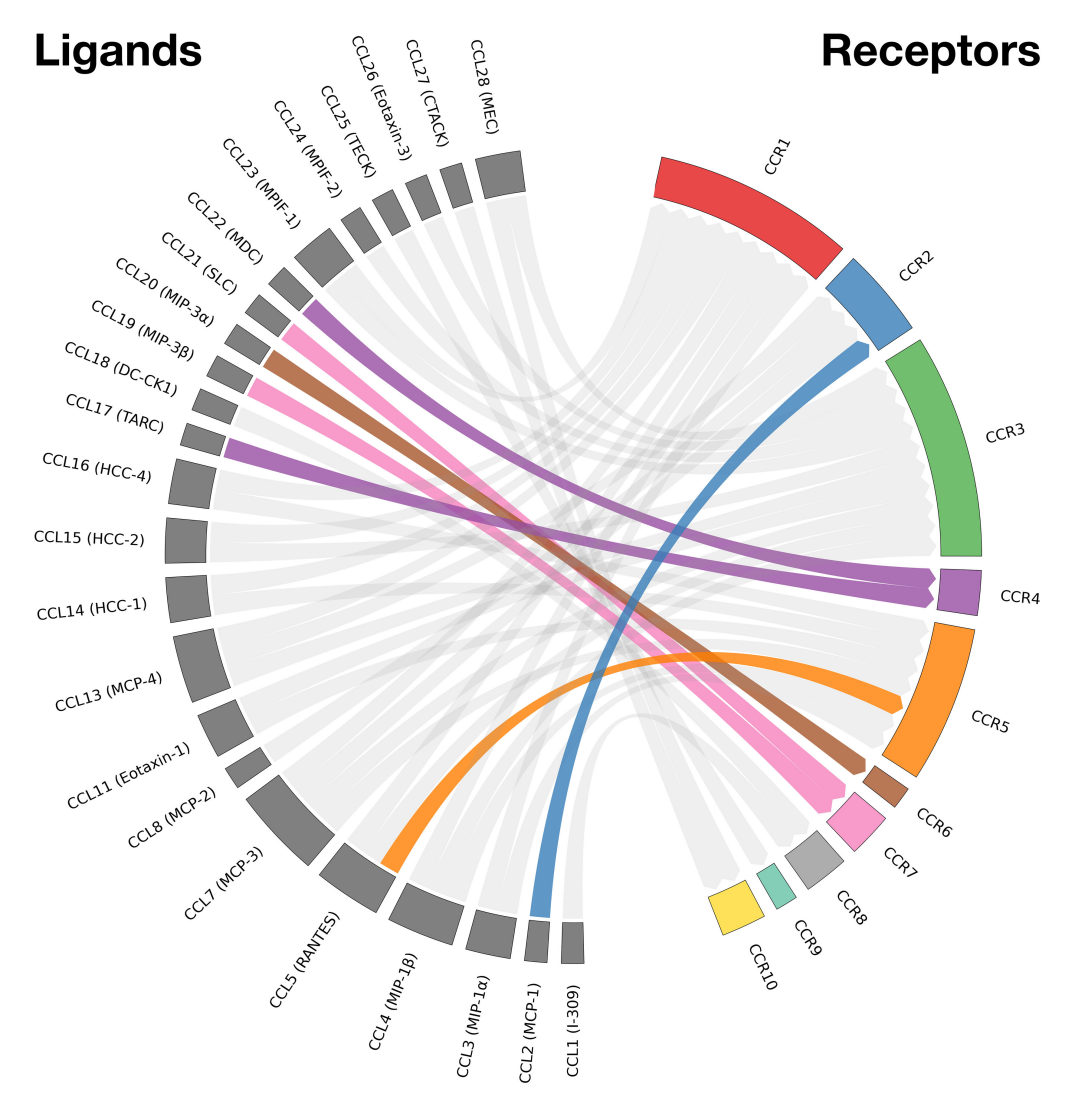
Chord diagram of known CC chemokine ligand-receptor interactions in humans. Binding relationships between human CC chemokines (CCL1-CCL28) and their corresponding receptors (CCR1-CCR10). Chemokines are shown on the left half of the circle, and their receptors on the right. Only human interactions are shown; mouse-specific ligands (e.g., CCL6, CCL9/10, and CCL12) are excluded. Only the five main CCR-CCL axes highlighted in this review (CCR2–CCL2, CCR5–CCL5, CCR4–CCL17/22, CCR6–CCL20, CCR7–CCL19/21) have been color coded. Adapted from Hughes et al. (10). Visualization generated with pyCirclize v1.9.1 in Python.

Among these various signaling groups is the CC chemokine family, constituting one of the four principal chemokine subfamilies (CXC, CC, XC, CX3C) distinguished by the arrangement of conserved cysteine residues near the N-terminus (9–11). Chemokines are small, secreted proteins that play essential roles in chemotaxis—the directed migration of immune cells such as monocytes/macrophages, T cells, and dendritic cells—through concentration gradients. CC chemokine receptor-ligand (CCR-CCL) interactions also play pivotal roles in immune cell polarization and the maintenance of immune homeostasis by directing leukocyte trafficking, although there have been implications of direct influence from chemokine signaling (9, 10). The CC chemokine family includes 27 known ligands (CCL1 to CCL28, with CCL9 and CCL10 representing the same gene product) and 10 known receptors (CCR1 to CCR10) (9, 10). Interactions within this predominantly non-exclusive ligand–receptor family (**Figure 1**)—with the exception of a few binding pairs—create an intricate signaling network leading to diverse downstream outcomes that extend beyond simple chemotaxis (9, 10, 12).

In the context of cancer, CC chemokines exert both anti-tumor and pro-tumor effects, though their influence is frequently skewed toward promoting tumor progression (13, 14). This pro-tumoral activity is mediated via several mechanisms, including the recruitment of immunosuppressive cell populations, support for cancer-associated fibroblasts (CAFs) and endothelial cell function, and the facilitation of angiogenesis, ECM remodeling, fibrosis, and metastasis (15–19). However, it is important to note that factors such as tumor stage or the immediate surrounding stromal context may cause the downstream outcomes of a specific CCR-CCL axis to differ significantly. Hypoxia, an inflammatory cytokine milieu, and/or abundant stromal signals, which are especially pronounced in PDAC, can influence the expression and activity of chemokines and their receptors, leading to context-dependent effects—discussed in more detail below (17, 20, 21). Several CCR-CCL axes—including but not limited to CCR2–CCL2, CCR5–CCL5, CCR4–CCL17/22, CCR6–CCL20, and CCR7–CCL19/21—have been identified as drivers in the recruitment and functional modulation of several different cell populations in PDAC (15, 19, 22–25).

## 2 Upstream sources and induction of CCR-CCL axes in PDAC

PDAC arises through a well-defined multistep progression involving the accumulation of genetic and epigenetic alterations in pancreatic ductal cells. The earliest and most common initiating event is an activating mutation in the KRAS oncogene, most frequently at codon 12, which is present in over 90% of low-grade PanIN lesions—the principal noninvasive precursors to PDAC (26, 27). The near-universal presence of KRAS mutations creates a permissive landscape for downstream chemokine signaling, driving persistent activation of signaling cascades including MAPK/ERK and PI3K/AKT, which collectively enhance NF-κB and HIF-1α transcriptional activity of chemokine genes (28–31). Mutant KRAS can also activate NF-κB via TAK1-mediated phosphorylation of IKKβ (32). KRAS-induced positive feedback loops, such as IL-6 and TGF-β secretion, activate JAK-STAT3 and SMAD pathways—also upstream to chemokine transcription and heavy stromal remodeling characteristic to PDAC (33–35). It is important to note here that while the neoplastic pancreatic ductal cells themselves may not produce all the chemokine populations—though they do produce some—the cytokines whose downstream expression is driven by KRAS oncogene mutations are responsible for coordinating chemokine upregulation via the previously described cascades (36). This genetic-epigenetic synergy between KRAS-mutant neoplastic cells, stromal cells, and dysregulated signaling pathways establishes a chemokine-rich milieu that drives PDAC progression, metastasis, and therapeutic resistance (**Table 1**).

**Table 1:** Core CCR-CCL axis function comparison in PDAC.

Receptor	Ligand	Cellular sources of ligands	Upstream pathways	Core functions	Targeted drugs
CCR2	CCL2	Basal-like tumor cells, CAFs (iCAFs, *FAP*^+^CAFs*)	NF-κB, STAT3, AP-1, HIF-1α, PPARδ	Monocyte/macrophage recruitment TAM and MDSC differentiation MMP upregulation	BMS-813160
CCR5	CCL5	Tumor cells, Granulocytes, Dendritic cells, TAMs, CD8^+^ T cells, CD4^+^ T cells, NK cells, Monocytes, CAFs*	NF-κB, STAT3, FOXP3, CD73, AP-1*	Monocyte/macrophage recruitment TAM and MDSC differentiation	BMS-813160 Maraviroc Leronlimab
CCR4	CCL17	Dendritic cells, M2a TAMs*	TSLP, CD40/CD40L*, STAT6*, GM-CSF*, *NF-*κB*, AP-1*	Treg and Th2 cell recruitment	FLX475
	CCL22	Dendritic cells, M2a TAMs*	TSLP, IL-1α, CD40/CD40L*, PU.1*, IL-4/IL-13*, *NF-*κB*, AP-1*		
CCR6	CCL20	Senescent tumor cells, TAMs, Dendritic cells*, Eosinophils*	NF-κB, IL-17B/IL-17RB, AP-1*, C/EBP*, SP1*, EGFR/Ras*	Treg/Th17 cell recruitment Tumor cell invasion/metastasis	PF-07054894 IDOR-1117-2520
CCR7	CCL19	TLS fibroblasts (rCAFs*)	Noncanonical p52/RelB NF-κB, LTβR, AP-1*	TLS formation Lymphatic invasion/LNMs Effector cell recruitment	CAP-100
	CCL21	TLS fibroblasts (rCAFs*)	Noncanonical p52/RelB NF-κB, *LT*βR*		

Cellular sources, intracellular pathways, core functions, and targeted drugs associated with each CCL type highlighted in this review. (*) Cell sources or upstream signals for which CCL induction has been reported only in non-PDAC contexts, under ambiguous circumstances, or where findings were inconclusive.

### 2.1 CCL2-specific expression origins

One prominent example of a chemokine that drives PDAC progression is CCL2, also known as monocyte chemoattractant protein-1 (MCP-1). CCL2 is abundantly secreted by basal-like tumor cells and CAFs—influenced upstream by signals from mutant KRAS oncogene-bearing tumor cells (37, 38). Among these CAF subtypes, iCAFs stand out in particular by being able to secrete CCL2 at levels exceeding 5000 pg/mL compared to a baseline lack of CCL2 expression in normal pancreatic tissues, placing it significantly higher than levels observed in breast (1300–1800 pg/mL) or prostate cancer (500–700 pg/mL) (39–42). Another significant CAF subtype—FAP^+^ CAFs—has also exhibited higher CCL2 activation in aggressive liver cancer models, and given their tightly shared desmoplastic traits, this mechanism is likely conserved in PDAC as well (35).

Intracellularly, several key transcription factors support CCL2 expression. The NF-κB and STAT3 pathways—the latter triggered by IL-6 trans-signaling across diverse tumor settings—serve as central drivers, not only for CCL2 but for a broad range of other chemokine axes as well (30, 34, 43). In basal-like PDAC tumor cells, BRD4-mediated c-Jun/AP-1 expression has also been implicated in sustaining CCL2 levels and its basal-like neoplastic state (38). Likewise, HIF-1α—a protein stabilized under the hypoxic conditions resulting from poorly developed vasculature and dense desmoplasia found in PDAC—further prompts CCL2 transcription (20, 31). The lipid-rich nature of the pancreas adds a unique layer, as sustained activation of PPARδs induces their binding to PPREs within the CCL2 promoter, amplifying CCL2 expression (44).

### 2.2 CCL5-specific expression origins

CCL5—also known as Regulated upon Activation, Normal T Cell Expressed and Secreted (RANTES)—is also a notably overactive chemokine in PDAC, and particularly pancreatic tumor cells, granulocytes, and dendritic cells (DCs) appear to be a major source of its production (36). However, additional evidence shows that CCL5 is also produced by tumor-associated macrophages (TAMs), CD8^+^ T cells, CD4^+^ T cells, NK cells, and monocytes, though not nearly at the level of the tumor cells, granulocytes, and DCs (36). Translational evidence from an ovarian cancer model also suggests CAFs can secrete CCL5 as well (45). Thus, understanding what drives CCL5 production in PDAC requires considering a relatively greater range of tumor and stromal cell populations.

Many of the transcription factors that mediate CCL5 expression in PDAC overlap with those regulating CCL2. Mentioned previously, both NF-κB and STAT3 are consistently observed as primary regulators of CCL5 in both general and pancreatic contexts (30, 46, 47). On the other hand, while c-Jun/AP-1-mediated CCL5 promotion has not been explicitly examined in the context of PDAC, it has been shown to be an upstream CCL5 activator in breast cancer settings, hinting at potential cross-tumor relevance (48). FOXP3—a transcription factor expressed by both regulatory T cells (Tregs) and certain cancer cells—can directly bind to the CCL5 promoter as well, enhancing its transcription and promoting the recruitment of CCR5^+^ Tregs into the tumor bed (16, 36, 49). Uniquely, CD73 has been shown to mediate CCL5-driven Treg recruitment via p38-STAT1 signaling (36). These findings imply a positive feedback system whereby CCL5 enhances Treg recruitment and establishes a Treg-FOXP3-CCL5-Treg signaling loop that maintains immunosuppression in PDAC. More targeted research is warranted before any definitive conclusions can be drawn.

### 2.3 CCL17/22-specific expression origins

CCL17 and CCL22—also known as thymus and activation-regulated chemokine (TARC) and macrophage-derived chemokine (MDC), respectively—are primarily secreted by DCs and macrophages in general settings (50, 51). In the context of PDAC, however, literature evidence remains focused on DC-derived CCL17/22 upregulation, particularly emphasizing DCs influenced by factors originating from tumor cells and fibroblasts (52, 53). On the other hand, macrophages/TAMs, while not explicitly mentioned, were implied via observations supporting CCL22 origins from immune cells of myeloid phenotype (52). Among macrophage subtypes, M2a phenotypes—largely known for their key role in fibrosis, a major element of PDAC desmoplasia, and characterized by their activation via Th2 cytokines IL-4 and IL-13—seem to be the most relevant in the context of CCL17 and CCL22 upregulation (54–57).

Several common intercellular signals drive the expression of CCL17 and CCL22, such as CD40L and TSLP via tumor cells and DCs, respectively (53, 58). However, while the CD40–CD40L pathway has been observed in follicular lymphoma, its relevance to PDAC remains hypothetical at best, warranting more tailored investigation (58). Despite shared inducers, the transcriptional routes of CCL17 and CCL22 can diverge. CCL17 depends primarily on IL-4-mediated STAT6 activation and GM-CSF signaling, although IL-4 and IL-13 has also shown simultaneous CCL22 upregulation (56, 59, 60, 126). In contrast, CCL22 is regulated by IL-1α and PU.1 (50, 52). This allows for context-dependent regulation, yet both chemokines are frequently co-expressed in tumor tissues, particularly within myeloid cell populations constitutively exhibiting both STAT6 and PU.1 (61, 62). NF-κB and AP-1 are likely central mediators of CCL17/22 transcription in PDAC as well. Although clear PDAC-specific evidence is lacking, studies from other disease contexts show that NF-κB and AP-1-driven expression of these chemokines is a common mechanism that likely stretches to PDAC (59, 63, 64).

### 2.4 CCL20-specific expression origins

CCL20—also known as macrophage inflammatory protein-3α (MIP-3α)—originates primarily from pancreatic tumor cells and M2 TAMs (65–67). Among tumor cell subtypes, however, senescent pancreatic tumor cells show the highest expression levels of CCL20, driven as part of the cytokine-rich senescence-associated secretory phenotype (SASP) (23). On the other hand, M2 TAMs typically rely on IL-4 signaling to induce CCL20, while other cell types—such as DCs and eosinophils—have also displayed CCL20 secretion; however, these observations have been restricted to non-PDAC settings, pointing out the need for pancreas-specific models (66, 67).

Multiple intracellular and intercellular pathways regulate CCL20 expression. NF-κB continues to be a key chemokine mediator in PDAC and plays a central role in promoting CCL20 levels (68). Additionally, the IL-17B–IL-17RB signaling axis has also demonstrated CCL20 upregulation in a murine model, where subsequent IL-17RB inhibition resulted in reduced metastasis, highlighting roles of CCR6–CCL20 in PDAC invasion (21). Separately, transcription factors AP-1, C/EBP, and SP1—likely upregulated via KRAS-driven p38 MAPK activity—have also demonstrated binding to CCL20 promoter regions (21, 69–71). Across several different cancer types—breast, colon, head and neck, and melanoma—EGFR-Ras-induced CCL20 expression has also been observed in murine models, suggesting this pathway may stretch to pancreatic contexts as well (72).

### 2.5 CCL19/21-specific expression origins

CCL19 and CCL21—also known as macrophage inflammatory protein-3β (MIP-3β) and secondary lymphoid tissue chemokine (SLC), respectively—are primarily produced by and active in fibroblast populations surrounding tumor-driven tertiary lymphoid structures (TLSs) and lymph vessels (73, 74). Among these different fibroblast populations, a scRNA-seq analysis on a stromal cell dataset in breast cancer models shows CCL19 and CCL21 production particularly enriched in the reticular-like CAF (rCAF) subset, compared to the seven other CAF subtypes classified in the study (75). These CAF subtypes, including rCAFs, have also been identified in PDAC as well (75).

The intracellular regulation of CCL19 and CCL21 expression is largely mediated by NF-κB signaling, especially through the noncanonical p52/RelB NF-κB pathway, which has been shown to constitutively drive CCL19 and CCL21 expression in pancreatic cancer (76). Other signaling elements, lymphotoxin-β receptor (LTβR) and AP-1—in coordination with NF-κB—also enhanced CCR7, CCL19, and CCL21 expression, supported by explicit preliminary PDAC evidence and/or translational models (73, 77–79).

## 3 CCR-CCL axes in PDAC

### 3.1 Immune cell recruitment and polarization

**Figure 2:**
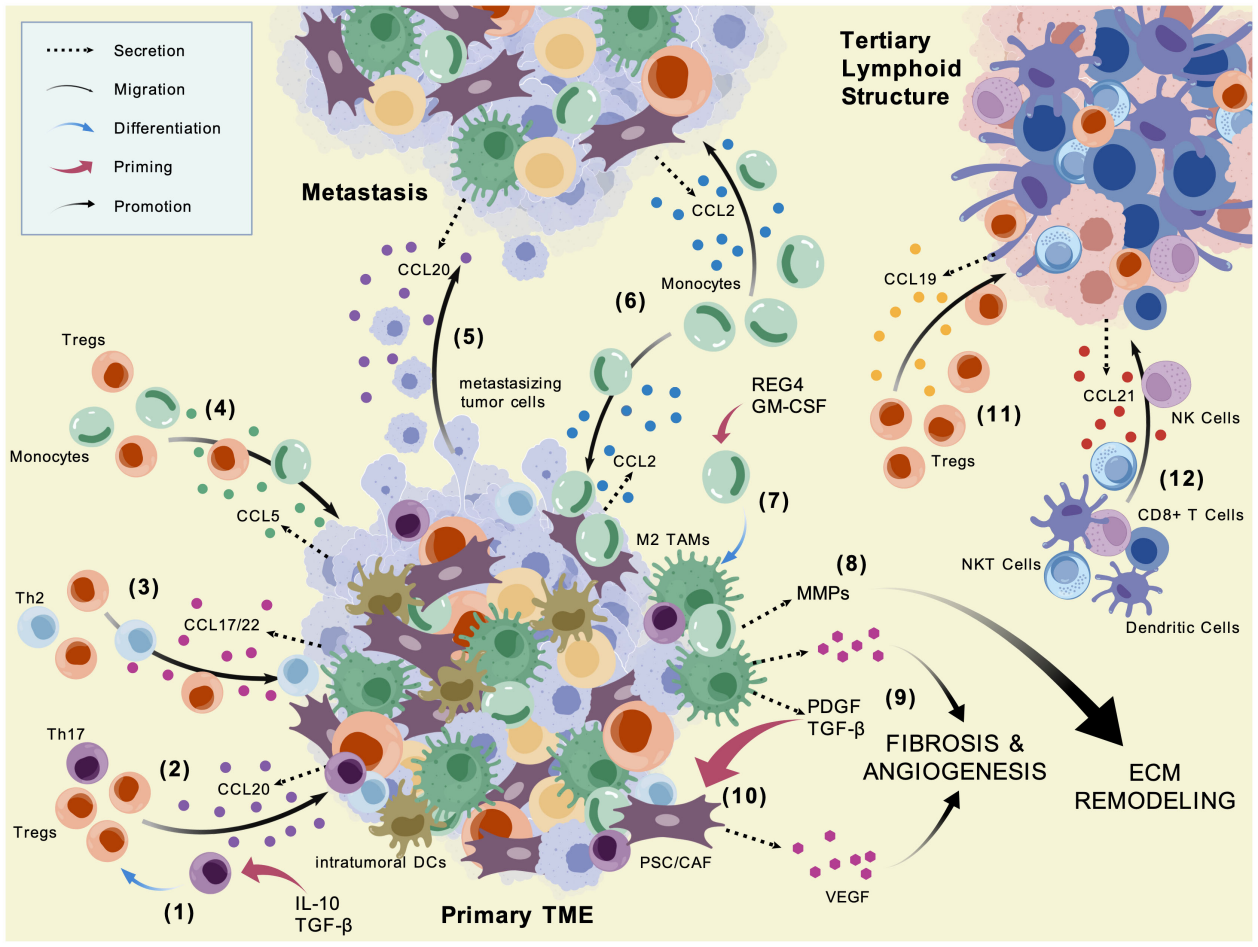
CC chemokine-mediated regulation of immune and stromal interactions in PDAC. This diagram highlights the roles of CC chemokines across the primary tumor microenvironment, metastatic sites, and tertiary lymphoid structures in PDAC, illustrating their involvement in cell recruitment, tumor progression, and immune organization. (1) Th17 cells differentiate into Tregs under the presence of immunosuppressive cytokines such as IL-10 and TGF-β; (2) CCL20 recruits Tregs and Th17 cells to the TME; (3) CCL17/22 recruits Tregs and Th2 cells to the TME; (4) CCL5 recruits Tregs and monocytes to the TME; (5) CCL20 mediates the migration of tumor cells to distant metastases; (6) CCL2 recruits monocytes/macrophages to the TME; (7) Monocytes differentiate into M2 TAMs under the presence of KRAS-mediated downstream signals GM-CSF and REG4; (8) MMPs secreted from monocytes and TAMs induce ECM degradation and remodeling; (9) VEGF signals from monocytes/macrophages and CAFs drive fibrosis and angiogenesis in the TME; (10) PDGF and TGF-β from M2 TAMs induce PSC/CAFs to secrete VEGF; (11) CCL19 recruits Tregs to the TLS; (12) CCL21 recruits CD8^+^ T cells, DCs, NK cells, and NKT cells to the TLS. Visualization generated with BioGDP.com (242).

In the PDAC TME, chemokines orchestrate the selective recruitment of immune cell subsets that predominantly reinforce immunosuppression (**Figure 2**). Monocyte infiltration—a hallmark of the PDAC TME—is primarily mediated by the CCR2–CCL2 and CCR5–CCL5 axes. Currently, PDAC literature mainly emphasizes CCR2–CCL2-induced monocyte chemotaxis, whereas studies on CCR5–CCL5-mediated monocyte recruitment in PDAC are lacking, although a breast cancer study has confirmed this function, suggesting a likely supplementary role in the context of PDAC (15, 80). However, while the CCR2–CCL2 axis remains restricted to monocytes—although under radiotherapy conditions, CCL2 has also been shown to recruit Tregs in HNSCC—CCR5–CCL5 exerts slightly broader immunoregulatory influence, also facilitating a portion of Treg infiltration and potentially recruiting CAFs as shown in a PDAC and ESCC model, respectively (16, 81, 82). General Treg accumulation, however, is most strongly associated with CCR4–CCL17/22 signaling, yet relevant studies examining PDAC-specific CCL17 remain absent, whereas the CCR4–CCL22 axis demonstrates a much clearer line of evidence—likely more focused on due to its binding superiority to CCR4 over CCL17 (52, 83–86). An *in vitro* investigation observing PBMCs in PaTu culture supernatents showed the level of migratory Tregs increase in cultures without CCL22 inhibition and return back to control levels in cultures with CCL22 inhibition (52). A separate murine pancreatic cancer model observed roughly 500 pg/mg CCL22 protein expression in a Panc02-OVA tumor compared to <20 pg/mg in the normal pancreatic tissue and CCR4 expression of tumor Tregs greater than or equal to twice of those of lymph nodes, spleens, lungs, and blood (84). Beyond CCR4, the CCR6–CCL20 axis may provide secondary support for Treg recruitment, while the CCR7–CCL19 pathway has been implicated in Treg chemotaxis in PDAC, based on findings translated from gastric cancer models (25, 87). Notably, although CCR4 is classically linked to Treg migration, recent patient-derived data in PDAC reveal an unconventional CCR4–CCL2 interaction that mediates monocytic myeloid-derived suppressor cell (M-MDSC) recruitment (88).

Although limited in effect, several CCR-CCL axes also contribute to the recruitment of anti-tumor immune cells in the PDAC TME. The CCR7–CCL19/21 axis—particularly the CCR7–CCL21 interaction—is strongly associated with the infiltration of effector populations, including CD8^+^ T cells, DCs, NK cells, and NKT cells, likely around lymphoid structures (74, 89). However, these cells are likely rendered dysfunctional by comparatively higher levels of immunoinhibitory signals and factors in the primary TME characteristic to PDAC—including, but not limited to, FOXP3, CTLA-4, IL-10, and TGF-β (90, 91). Additional CCR-CCL interactions offer only minor contributions to anti-tumor infiltration: the CCR4–CCL17/22 axis can attract Th2 cells, though this is likely outweighed by its dominant role in Treg trafficking; similarly, the CCR6–CCL20 axis may recruit Th17 cells under inflammatory conditions, but this potential is suppressed by the immunologically ‘cold’ nature of PDAC (87, 92, 93).

Beyond recruitment, CCR-CCL signaling axes play key roles in shaping the immunosuppressive characteristics of the PDAC TME by driving the polarization of both myeloid and lymphoid cells. M2 macrophage polarization is promoted directly by mutant KRAS-mediated downstream signals, such as elevated levels of GM-CSF and REG4, which converge onto an AKT-mediated cascade (94, 95). MDSC polarization—which often produces overlapping results with TAMs—appears to rely mainly on GM-CSF signaling (96). Among the previously covered chemokine axes, CCR2–CCL2 and CCR5–CCL5 are implicated in inducing both M-MDSC and M2 TAM phenotypes, largely in part due to their function in monocyte recruitment—the shared progenitor of M-MDSCs and TAMs (97, 98). Signaling axes CCR6–CCL20 and CCR7–CCL19/21 have also demonstrated M2-polarizing effects via either explicit observations in PDAC or translational evidence from other solid cancer studies (23, 99). Wu et al. showed CCL20^+^ ductal 2 cells to be positively associated with the M2 phenotype by inducing high CD206 and TGF-β expression—both of which are typical M2 products—in a macrophage cell line through CCL20 treatment (23). The CCR6–CCL20 axis has also shown context-dependent effects on Treg plasticity. In the context of surrounding tissue inflammation, CCR6 expression in conjunction with RORγt^+^ transcriptional programming can facilitate the conversion of iTregs into Th17 cells, suggesting a dynamic immunomodulatory potential, although whether RORγt^+^ Tregs represent a population with reduced suppressive capacity remains unclear and appears to depend on additional contextual factors (91, 100, 101). However, as the TME advances toward a more immunosuppressive cytokine profile—marked by high levels of IL-10 and TGF-β—surrounding signals contribute to Treg stabilization, effectively suppressing Th17 differentiation and reinforcing the dominance of regulatory phenotypes (91).

### 3.2 TME remodeling

#### 3.2.1 Angiogenesis and fibrosis

Angiogenesis and fibrosis are hallmarks of TME remodeling, and CCR-CCL signaling axes play a central supporting role in inducing VEGF-driven vascular growth. M2 TAMs—recruited and activated via CCL2—serve as major secretors of VEGF and other angiogenic factors like FGF, as shown in pancreatic cancer models (15, 102, 103). Under this logic, the other previously mentioned chemokine axes—CCR5–CCL5, CCR4–CCL17/22, CCR6–CCL20, and CCR7–CCL19/21—may further contribute to VEGF secretion through their variable influences on macrophage polarization and/or recruitment, though direct causal evidence remains limited (23, 24, 98, 104). In parallel, the CCR10–CCL28 axis mediates the migration of PSCs into the TME, where the CCL2-induced M2 TAMs also secrete signals like PDGF and TGF-β, which activate these recruited PSCs, which have been known to be the primary CAF precursor (17, 105). In recent years, however, the notion of PSC dominance among CAF precursor populations has been challenged as new studies demonstrate PSCs as a rather minor subset among other distinct precursor populations such as Gli1^+^ cells (106, 107). Nevertheless, PSCs and CAFs have both demonstrated VEGF production—particularly in response to HIF-1α presence as a result of chronic hypoxia—and secretion of fibrotic components such as collagen, reflecting their significant role as key mediators of TME fibrosis (108, 109).

Interestingly, CCR10–CCL28-mediated PSC migration to the PDAC TME was shown to be inflammation-driven, implying that PSCs tend to accumulate in peritumoral regions compared to central desmoplastic zones, which are characterized by a more anti-inflammatory cytokine milieu—an inference supported by spatial distribution analysis (17, 110, 111, 224). Peritumoral aggregation of PSCs and CAFs may represent a key driver of outward tumor expansion, as these cells prime the surrounding stroma for invasion by promoting fibrotic and angiogenic remodeling that facilitates tumor cell spread—an interconnected mechanism that warrants tailored investigation. Notably, MDSCs have also been linked to VEGF production in various tumor models, linking MDSC chemotaxis and polarization contributing axes—predominantly CCR2–CCL2—as upstream influencers of this observation, although studies connecting CC chemokine axes to MDSC-mediated VEGF production in PDAC specifically remain unexplored (97, 98, 112). The same literature pattern is seen in Tregs, suggesting that the CCR4–CCL17/22 axis, and to a lesser extent CCR5–CCL5 or CCR10–CCL28, may significantly influence angiogenesis by facilitating the recruitment of Tregs, although VEGF secretion has not been specified (16, 84, 113).

Beyond immune cell-driven mechanisms, tumor cells themselves have shown VEGF secretion in response to CCL5 via bone cancer models (114, 115). However, this pathway remains to be validated in the context of PDAC. In contrast, CCR7–CCL21 and VEGF-C expression have shown high correlation explicitly in PDAC and have been implicated in promoting both angiogenesis and lymphangiogenesis—supported by its association with elevated microvessel and lymphatic vessel densities, although concrete mechanistic studies have yet to happen (19, 116). Despite these insights, PDAC-specific evidence for CCR-CCL-driven angiogenesis and fibrosis remains preliminary, where much literature draws from data obtained in broader tumor studies and predominantly reveals correlative findings rather than mechanistic data, thus highlighting the need for more targeted research in PDAC.

#### 3.2.2 Matrix metalloproteinases

MMPs are key mediators of ECM degradation in PDAC, regulated in part by CC chemokine signaling. In one study, high incidence rates of MMP2, MMP7, and MMP9 expression (79.31%, 55.17%, 24.13%, respectively) among 29 patients have been observed in the PDAC stroma compared to normal tissue regions—all of which demonstrated a relative lack of these same signals (3.45%, 6.90%, 0%, respectively) (117). A different investigation utilizing single-cell RNA sequencing analysis revealed that CCR2/CCL2^+^ macrophages directly facilitated MMP9 promotion in PDAC (118). Likewise, CD40-mediated CCL2 and IFN-γ upregulation promoted the recruitment of Ly6C^+^ inflammatory monocytes, which subsequently secreted MMP10, MMP12, and MMP13 (119). Given that IFN-γ is a well-characterized pro-inflammatory cytokine, this observation may be especially relevant to peritumoral regions as well, where the surrounding cytokine milieu is comparatively more inflammatory and may require ECM breakdown and subsequent immunosuppressive conditioning prior to outward tumor invasion—similar to the potential peritumoral PSC/CAF aggregation mechanism described previously. This proposed regional relationship remains to be validated. Separately, an *in vitro* PANC-1 cell culture investigation observed CCL20 to promote the upregulation of MMP9 and a subsequent dose-dependent increase in tumor cell invasion. This effect was abrogated in the presence of an anti-CCR6 or anti-MMP9 antibody by similar degrees to 37% and 35% of control, respectively (65). In a different *in vitro* PANC-1 experiment, Cui et al. found a correlation between CCL21 and MMP9 via western blot analysis, indicating a role of the CCR7–CCL21 axis in ECM degradation (120). Beyond these observations, additional PDAC-specific experiments investigating CCR-CCL-induced downstream MMP production remain scarce; however, many translational liver cancer and HCC models have shown several cases of this general pathway—particularly highlighting MMP2 and MMP9 over other MMP types (121–124). More broadly, CCR-CCL driven MMP induction has also been reported in lung, ovarian, prostate, breast, and colon cancer studies (125–129). But based on current literature consensus, the CXCR4–CXCL12 axis seems more widely supported as the primary mediator of MMP induction in PDAC (130–132). However, because this axis lies outside the scope of the present review, it will not be discussed in further detail here. Taken together, while there is a growing pool of preliminary evidence suggesting that CCR-CCL signaling causes MMP upregulation in PDAC, this field lacks mechanistic proof. Most studies remain associative, leaving a critical gap in our understanding of PDAC ECM remodeling, which holds eventual implications for metastasis as well, a topic explored in more detail below.

#### 3.2.3 Tertiary lymphoid structures

The formation of TLSs within the PDAC TME is increasingly recognized as a critical mediator of anti-tumor immunity. TLSs are immune cell aggregates that form through inflammatory signals that arise during diseases such as cancer (133). In general, the presence of TLS has been highly associated with improved prognosis and better survival rates across several cancer models, particularly in concert with immune CPIs—which have been ineffective as PDAC monotherapies (3, 134). The CXCR5–CXCL13 and CCR7–CCL19/21 axes play key roles in TLS formation, serving as primary promoters of B and T lymphocyte infiltration in coordination with LTi and LTo cells triggered by IL-7 and LTβR–LTα1β2 signals during the early stages of TLS formation across GI cancers, including PDAC (135, 136). The roles of CCL19 and CCL21 in particular appear to be concentrated in DC and T cell activation and migration (136–138). In addition, the CCR6–CCL20 axis—although not as mechanistically investigated in literature—has shown potential in TLS development and maintenance due to its role in effector cell migration, including Th17 infiltration in inflammatory settings as well as B cells and DCs in other cancer models (87, 139, 140). However, a clear association with TLSs is never made in these studies, and the specific subset of the recruited immune cells should be carefully considered before making any hypotheses. Nevertheless, Pushpamali et al. showed upregulation of several signals in tumors containing TLSs, one of which was CCL20, suggesting a larger than previously understood role of the CCR6–CCL20 axis in TLS functioning (141). TLS presence, however, remains generally uncommon in PDAC patients. A study analyzing the prognoses of PDAC TLSs reported that improved outcomes were mostly correlated with an intratumoral subset of TLSs, observed in only around 15-20% of patients (142). Together, these findings underscore the roles of CC chemokines in TLS promotion, while highlighting the potential strategy of TLS induction as a therapy to overcome PDAC.

### 3.3 Metastasis and PMN formation

In PDAC, TAMs contribute to metastatic progression through CCR-CCL axis-mediated recruitment and polarization, while MMPs, which often intersect with TAM-driven pathways, facilitate invasion by degrading the ECM. Sanford et al., via an experiment dealing with the investigational drug PF-04136309, demonstrated that it is monocytes after differentiation into TAMs—rather than the initial CCR2^+^ inflammatory monocytes—that primarily mediate immunosuppression (15). In this study, PF-04136309 reduced TAM levels in the pre-metastatic liver, whereas—in a separate phase 1b trial using the same investigational drug PF-04136309 (NCT02732938)—TAM levels in the primary tumor body showed minimal or ambiguous change, a discrepancy likely attributable to the more pronounced desmoplastic and immunosuppressed stroma in the primary tumor compared to the PMN, which is only in the preparatory stages of tumor cultivation (3, 15, 143). While this phase 1b trial did not report detailed efficacy endpoints on metastasis due to its early-stage nature, the available evidence suggests that CCR2–CCL2 contributes to metastatic progression primarily through TAM accumulation at the PMN rather than at the primary tumor site, a mechanism that warrants further investigation (15, 143). Supporting this idea, two separate *in vitro* pancreatic models have demonstrated CCL20 dose-dependent invasion of collagen by PANC-1 cells at 100ng/mL, while a different ex vivo investigation showed M2 TAMs upregulating CCL20 by roughly three fold in metastatic compared to non-metastatic clinical PDAC tissue samples, promoting EMT and tumor cell migration (65, 67, 144). This may suggest that CCR2–CCL2-driven PMN TAM proliferation is a prerequisite for CCL20-mediated early metastasis, enabling both initial tumor cell recruitment and MMP9 promotion, which helps establish a permissive environment for tumor dissemination beyond the primary tumor mass (65, 67). CCR2^+^ macrophages have specifically been shown to induce MMP9 in pancreatic lymph node metastases, a pathway that likely stretches to other pancreatic metastatic sites (117). Although direct indications of CCR-CCL-driven MMP expression in PDAC remain rare, extensive data from other cancer models support this relationship. In nasopharyngeal carcinoma, CCR2–CCL2 has been shown to upregulate MMP9 and MMP2 (145). The CCR5–CCL5 signaling axis exhibited MMP9 promotion via pulmonary mesenchymal cells in lung cancer and through PLC, PKCδ, and NF-κB in oral cancer (146, 147). And the CCR4–CCL17 axis has demonstrated MMP13 activation via ERK1/2 signaling in bladder cancer (148). These cascades serve as potential therapeutic targets in PDAC metastasis, but their exact roles in the pancreatic setting remain unclear.

Currently, despite additional literature around CCR-CCL-driven PDAC-specific organ metastasis remaining narrow, other correlative cancer models still underscore the necessity for further research in this field. For example, studies of CCR5–CCL5 in pancreatic tumor cell invasion are largely confined to *in vitro* correlative models (22, 149). In contrast, breast cancer—particularly TNBC, which shares many immunosuppressive and mesenchymal features with PDAC—offers more extensive insights (150). TNBC cell-secreted IL-6 showed LEC priming in pre-metastatic organs to promote CCL5 levels in coordination with VEGF—via a pSTAT3/p-cJun/pATF-2 ternary complex—thereby recruiting CCR5^+^ tumor cells to developing PMNs (151). LncSNHG5 and IGF2BP2 further stabilize these niches by supporting ZNF281—a CCL2/5 transcription factor— and activating the p38-MAPK signaling axis in HUVECs, which upregulates angiogenesis and vascular permeability (152). In a separate TNBC model, Chen et al. identified a CCR5-driven mechanism linking EMT and metastasis via HIF-1α, YAP1, ZEB1/2, and β-catenin (153). Beyond CCR5, CCR4^+^ Tregs showed breast cancer lung metastasis promotion via βGBP-driven NK cell suppression (154). Gastric cancer models showcasing peritoneal spread—which are particularly relevant given that peritoneal spread is the second most common metastatic site in PDAC after the liver—also show proof for CCR-CCL axis involvement in peritoneal invasion (155, 156). *In vitro* gastric cancer studies have shown upregulation of CCL5 among many other signals leads to peritoneal metastasis, and ex vivo human analyses confirm strong correlative patterns (157, 158). Another experiment uniquely reported CCR4 expression in gastric cancer peritoneal metastasis exhibiting preferential aggregation toward omental milky spots—an important consideration in potential future PDAC investigations (159). Separately, CCL17 demonstrated significant upregulation as early as 10 weeks before micrometastatic formation in a melanoma model, suggesting that the CCR4–CCL17 axis plays a part in early PMN establishment (160). These findings across several different cancer models may offer translatable frameworks when investigating CCR-CCL-driven metastasis in the context of PDAC.

Multiple CCR-CCL axes have been implicated in promoting PDAC LNM, acting via both tumor-intrinsic and immune-mediated mechanisms. LNM prevalence in PDAC stands at roughly 65-85% and serves as a strong prognostic factor that correlates with greatly reduced OS compared to node-free patients (161–163). The CCR7 signaling axis has been identified as a primary driver of LNMs in PDAC. Elevated CCR7 levels in pancreatic tumor cells with nodal metastases relative to normal tissues have also been confirmed via ex vivo observational analyses (19, 164). In particular, cells expressing CD133—a commonly associated marker of CSCs—showed significantly increased CCR7 expression levels (164). A different study examining CD133 and not CCR7 also separately verified CD133 as a key distinguishing element between metastatic and non-metastatic pancreatic cancer cell lines, heavily indicating a link between CD133 and metastasis (165). This relationship between CD133 and CCR7 remains to be elucidated. Interestingly, CCR7 upregulation was found absent in PDAC liver or lung metastases, suggesting an exclusivity to lymphatic spread (166). However, this same observation was absent in a different model featuring liver metastases in colorectal cancer, indicating a context-dependent nuance (167). Other CCR-CCL axes have shown signs of involvement in LNM as well. One study revealed high levels of CCL2^+^ CCR2^+^ macrophages in PDAC LNMs, which appeared to recruit Tregs and dysfunctional CD8^+^ T cells, likely cultivating the same suppressive setting observed in the primary TME (117). The CCR5–CCL5 axis showed strong correlation with LNM in a gastric cancer model and appeared to induce metastasis by skewing the Th1/Th2 immune ratio in favor of an immunosuppressive Th2 phenotype, although further mechanistic examination is warranted (168). These observations highlight CC chemokine axes—particularly CCR7—as key drivers of nodal metastases in PDAC.

## 4 Chemokine mediated downstream immune evasion

Several immune evasion pathways are influenced by chemokine-mediated regulatory cell populations in the PDAC microenvironment. Among these immunosuppressive populations, Tregs are a key player in helping maintain an immunologically “cold” setting by suppressing effector immune responses via paracrine signaling and transcriptional modulation involving proteins like CTLA-4, PD-1, IL-10, TGF-β, and FOXP3, among others (83, 91, 169). In PDAC and colon cancer investigations, the CCR5–CCL5 signaling axis seems to have a large influence over these proteins (98, 170). In another model examining various cancer types, upregulated glycolytic activity in tumor-infiltrating Tregs has been linked to the CCR6–CCL20 axis, which has also been correlated with reduced OS (171). The CCR4–CCL22 axis has also demonstrated a role in Treg activation, distinct from Treg recruitment, via inducing DC-Treg interactions in nodal settings (172). Interestingly, although CCL22-deficient mice showed elevated anti-tumor immune responses, Treg infiltration remained comparable to wild-type controls, suggesting that CCR4–CCL22 may perform a larger than known role in Treg activation versus recruitment, likely compensated by alternative cascades (172). In parallel, M2 TAMs and MDSCs represent additional chemokine-driven immunosuppressive populations that synergize with Tregs. M2 TAMs produce inhibitory mediators like IL-10, TGF-β, PD-L1, and IL-6—which suppress effector cell function and support Treg activity—and MDSCs exert many of these same roles also via ROS, NO, and ARG1 production (173–176). M2 TAMs and MDSCs further back tumor progression by secreting growth factors VEGF and PDGF, working in concert with CAFs to support demoplasia (102, 177, 178). By producing dense stroma components—primarily fibronectin and type I collagen—CAFs construct physical and biochemical barriers that impair vascular perfusion, drug delivery, and nearby inflammatory signals (179–181). Consequently, the PDAC TME develops into a highly integrated network between different interdependent cell populations. M2 TAMs and Tregs participate in extensive crosstalk—where autocrine signaling and positive feedback mechanisms foster mutual reinforcement—and MDSCs possess the capacity to differentiate into TAM-like cells under hypoxic conditions (182, 183). Separately, CAFs have also been shown to assist in M2-like polarization of monocytes as well (184).

Th2 and Th17 cells also play important roles in the immune landscape of PDAC, albeit in a more nuanced and functionally plastic manner. Under inflammatory conditions, the CCR6–CCL20 axis has the ability to recruit Th17 cells, although to a lesser extent compared to other immune cell types (87). The current body of literature represents a mix of both pro-tumorigenic and anti-tumorigenic functions of Th17 cells. In PDAC-specific models, Th17 cells exert pro-tumor development modulation via NETs and control of gut microbiota-tumor interactions via IL-17 (185, 186). A different subset of IL-17-producing cells, dubbed Tc17 cells, has also shown pro-tumor outcomes via CD8^+^ T cell suppression and iCAF differentiation (187). On the other hand, broader tumor models exhibit Th17-mediated anti-tumor immunity through DC induction and/or differentiation into Th1-like or Th17/Th1 hybrid phenotypes, strengthening cytotoxic immune responses (188, 189). Likewise, elevated levels of IL-6 in concert with TGF-β leading to Th17 cell differentiation over Treg development resulted in delayed tumor growth and improved survival in a murine pancreatic model (91, 190). Taken together, the dual nature of Th17 responses underscores their plasticity in PDAC, while in contrast, Th2 cells—recruited via CCR4–CCL17/22 signaling—exhibit a functional bias toward tumor-promoting effects rather than a balance, one of which is M2 macrophage polarization through IL-4 and IL-13 expression (105, 191). IL-4 has also clearly demonstrated a dose-dependent promoting role in tumor proliferation and metastasis within pancreatic cancer (192, 193). On the contrary, findings by Jacenik et al. revealed that Th2 transduction resulted in eosinophil anti-tumor activity—partially driven by Th2 cell secreted IL-5—in pancreatic cancer lines (194). This IL-5-secreting Th2 subset suggests a unique immunological profile that warrants more investigation to clarify its relevance in PDAC. Th2 and Th17 cells thus contribute to PDAC from several angles, where pro-tumorigenic outcomes remain dominant, although context-specific anti-tumor exceptions remain deserving of attention.

## 5 Therapeutic targeting of CCR-CCL axes and its patient outcomes

Despite several decades of research, the current PDAC standard of care remains largely limited. Upon diagnosis, treatment typically starts with surgical resection—when feasible—and is paired with neoadjuvant and/or adjuvant chemotherapy or radiotherapy (195). Depending on the patient’s condition, first-line or neoadjuvant/adjuvant chemotherapy generally entails either a gemcitabine and nab-paclitaxel combination or a 5-fluorouracil (5-FU) based regimen among other options (195). Since its approval in 1995, gemcitabine has acted as the standard of care over 5-FU therapy, and nab-paclitaxel was introduced in 2013 as a combinatorial partner to enhance efficacy (196–198). FOLFIRINOX—a regimen comprising 5-FU, leucovorin, irinotecan, and oxaliplatin—entered clinical practice following a pivotal 2011 study as the first 5-FU-based regimen to demonstrate comparable outcomes, followed shortly thereafter by the 2015 approval of Onivyde (199, 200). NALIRIFOX, another 5-FU-based chemotherapy approved more recently in early 2024, has shown improved mOS (11.1 vs 9.2 months) with a virtually identical AE profile when placed head to head with a gemcitabine-nab-paclitaxel treatment arm (201–203). In addition to standard therapies, biomarker-driven treatments such as olaparib, pembrolizumab, zenocutuzumab, larotrectinib, and entrectinib target select PDAC patient subsets (195, 204–208). However, only a small fraction of the total population meet the given criteria, typically applicable to less than 5% or—in some cases—roughly 1% of the total patient population (209–213). Conventional chemotherapy and radiotherapy also face challenges, such as consequent chemokine upregulation—particularly CCL2 among other chemokine types—which limit the treatments’ efficacy (81, 214, 215). While 5-FU-based chemotherapies have not yet demonstrated similar outcomes in PDAC, preliminary evidence suggests that CCL upregulation via the STING pathway is plausible and may lead to disease relapse (216–218). Given these shortcomings, recent therapeutic strategies have begun to explore combinatorial approaches that integrate standard chemotherapy or radiotherapy with immune-based interventions.

Among these immune-based targets, CCR-CCL inhibition has emerged as a promising therapeutic avenue. While no CCR-CCL targeting drugs have been approved for PDAC yet, several mid-stage drug candidates are currently under clinical trials to address this therapeutic gap (**Table 2**). BMS-813160—a small-molecule dual antagonist of CCR2 and CCR5—remains the most explored in the context of PDAC. Although the efficacy results of two trials (NCT03184870, NCT03767582) investigating BMS-813160 in PDAC have yet to be released, a third study (NCT03496662) reported promising outcomes in BR (ORR 42%, mPFS 11.9 mo, mOS 18.2 mo) and LA PDAC (ORR 20%, mPFS 14.7 mo, mOS 17 mo), representing comparable to moderate improvements relative to historical benchmarks, particularly in LA PDAC (219–221). Leronlimab—an IgG4 monoclonal antibody for CCR5—also demonstrates translational potential in PDAC via activity in TNBC-centered trials (150). A pooled analysis of three mTNBC studies (NCT03838367, NCT04313075, NCT04504942) showed that higher doses (525-700mg), compared to lower doses (350-525mg), resulted in >75% improvement in mPFS and mOS, with outcomes reaching 6.1 months and over 12 months, respectively (222). CytoDyn, the developers of leronlimab, has also announced through a press release that the high-dose patient arm showed subsequent PD-L1 upregulation—suggesting a potential combination therapy with CPIs—and that long-term follow-up observed >36 months of OS in some cases (223, 224). Maraviroc—a small-molecule CCR5 antagonist currently only approved for HIV—has also emerged as a therapeutic candidate, supported by extensive preclinical PDAC models (22, 149, 225). In the context of clinical trials, however, maraviroc has been primarily tested in CRC (NCT01736813, NCT03274804), although a separate trial (NCT04721301) named LUMINESCENCE has assessed it in PDAC (226, 227). Notably, results for the LUMINESCENCE trial have yet to be released despite its completion in March 2023.

**Table 2:** Clinical stage drug candidates targeting the CCR-CCL signaling network.

Target	Drug name	NCT/ISRCTN	Phase	Condition(s)	Status	Results
CCR2 /CCR5	BMS-813160	NCT01049165	1	Healthy	Completed Nov 2011	–
		NCT03184870	1b/2	mCRC, mPDAC	Completed Jun 2023	–
		NCT03767582	1/2	LA PDAC	Completed Feb 2025	Phase 1 portion determined a RP2D of 300mg PO BID; Phase 2 results have yet to be released (243)
		NCT03496662	1/2	BR/LA PDAC	Completed Jul 2024	BR PDAC showed 42% ORR, 11.9 mos mPFS, 18.2 mos mOS; LA PDAC showed 20% ORR, 14.7 mos mPFS, 17 mos mOS (219)
		NCT04123379	2	NSCLC, HCC	Active	–
CCR5	Maraviroc	NCT01736813	1	mCRC	Completed Sep 2014	7.32 mos mOS (226)
		NCT03274804	1	MSS CRC	Completed Mar 2020	5.3% ORR, 2.1 mos mPFS, 9.8 mos mOS (227)
		NCT04721301	1	mCRC, mPDAC	Completed Mar 2023	–
	Leronlimab	NCT03838367	1/2	mTNBC	Terminated*	The overall pooled cohort (n=28) showed 3.8 mos mPFS, 6.6 mos mOS; the higher dose subgroup (n=19) showed 6.1 mos mPFS and >12 mos mOS (222)
		NCT04313075	Expanded Access	mTNBC	N/A	
		NCT04504942	2	LA/metastatic solid tumors	Active	
		NCT06699836	2	MSS CRC	Recruiting	–
CCR4	FLX475 (Tivumecirnon)	NCT03674567	1b/2	Advanced Cancer	Completed Dec 2024	The entire patient cohort (n=35) showed 26% ORR; low PD-L1 subgroup (n=16) showed 31% ORR; the high PD-L1 subgroup (n=4) showed 50% ORR (228)
		NCT04768686	2	Advanced/metastatic GC	Completed Aug 2024	Cohort 1 (EBV-, n=10) showed no ORR, only stable disease was achieved; Cohort 2 (EBV+, n=10) showed a 60% ORR, 10.4 mos mPFS, and no mOS was reached (230)
CCR6	PF-07054894	NCT04388878	1	Healthy	Completed Jun 2022	–
		NCT06327880	1	Healthy	Completed Jul 2024	–
		NCT07009353	1	Healthy	Completed Sep 2025	–
	IDOR-1117-2520	ISRCTN28892128	1	Healthy	Deferred**	–
CCR7	CAP-100	NCT04704323	1	CLL, SLL	Recruiting	–

Overview of therapeutic agents in clinical development as of December 2025 targeting the CCR-CCL signaling network. The table lists molecular targets, drug names, trial identifiers (NCT/ISRCTN), phases, conditions under investigation (including cancer and select nonmalignant diseases), trial statuses, and trial results, where applicable. (*) Terminated due to business reasons; (**) Trial has been completed as of April 6, 2024, but a publication of results has been deferred.

Shifting toward less-focused-on axes, FLX475—a small-molecule CCR4 antagonist also referred to as tivumecirnon—has been clinically tested in CPI-naive NSCLC, CPI-experienced HNSCC, and mGC (NCT03674567, NCT04768686). Across these studies, FLX475 exhibited greater efficacy in tumors characterized by high PD-L1 expression, HPV positivity, and/or EBV positivity, though the mid-stage nature of the trials and small sample sizes require the findings be assessed conservatively (228–230). PDAC-specific studies will be necessary to support clinical translation of FLX475, given the pro-tumor roles of CCR4 in the pancreatic setting (24). Clinical targeting of CCR6 and CCR7 remains even more scarce. CCR6 antagonists, PF-07054894 and IDOR-1117-2520, are both under early-stage clinical investigation, primarily focused on evaluating safety and toxicity in healthy individuals (NCT07009353, NCT04388878, NCT06327880, ISRCTN28892128). Preclinical studies, however, have demonstrated that PF-07054894 and IDOR-1117-2520-mediated blockade can reduce immune cell infiltration into tissues in autoimmune models, indicating a mechanism that may be viable in tumors but warrants more data (231, 232). CAP-100—an anti-CCR7 monoclonal antibody—is currently under phase 1 evaluation for CLL and SLL (NCT04704323), indications in which CCR7 plays a relatively well-defined role in disease progression (233). Although CCR7 expression in PDAC is more variable, preliminary findings suggest that CCR7 blockade may exert anti-LNM effects in pancreatic cancer, though again, the approach should be pursued with caution (234). These efforts highlight the growing interest in CCR-CCL axis targeting for drug development in PDAC.

## 6 Discussion

Through this review, we have sought to provide a comprehensive look into our current understanding of CC chemokine functions in the context of PDAC. Generally, literature evidence has pointed toward CCR-CCL axes as central drivers of immunosuppression in the PDAC TME—although there have been nuanced cases of inflammatory, anti-tumor outcomes—and enhance overall immune evasion not only via chemotaxis and polarization of regulatory cell types, but also via ECM remodeling and metastasis promotion through downstream cascades. While this review predominantly examines select signaling pairs, it is important to note that other CC chemokine pathways that were only briefly covered (CCR10–CCL28 and CCR4–CCL2) or were not mentioned at all (CCR8–CCL1, CCR1–CCL3/15) also play relevant functions likely in subtler ways (17, 88, 235–237). Likewise, the CXC chemokine family—often functioning in parallel or in coordination with CC chemokines—introduces another added layer of intricacy and was not covered in detail here in order to prioritize greater depth and synthesis over breadth (238). Insights based in this field should, if not already, take these other important elements into account as well.

Another major factor to consider is the differences the PDAC TME has with other tumor types when analyzing their translational value to PDAC-specific applications. As frequently mentioned throughout this review, the dense desmoplastic stroma present within PDAC is a key differentiating factor that imposes restrictions on immune cell infiltration. As such, the PDAC TME exhibits a comparatively greater degree of immune suppression, particularly via TAMs and MDSCs because of its high reliance on monocyte chemotaxis through the CCR2–CCL2 axis (15). The dependence of PDAC on myeloid cells also provides additional nuance when compared to tumor types that have a heavier dependence on lymphoid cell populations, such as lung cancer or melanoma (239, 240). Furthermore, discrepancies in current literature—such as the absence of CCR7 in PDAC liver and lung metastases as opposed to its presence in CRC liver metastases—also require caution when extrapolating findings investigated only in non-PDAC tumors (167).

Looking ahead, despite rising recognition and research, this particular field still possesses several knowledge gaps that may cause hesitancy in its therapeutic applicability, as portrayed by the apparent lack of clinical trials explicitly in PDAC among the already small number of relevant drugs covered. A recurring theme that showed potential for future investigation is using spatial transcriptomics to analyze around peritumoral regions of PDAC in the context of inflammatory markers. In several instances, cross-paper analysis suggested that inflammatory signals promote chemokine axes to recruit immune cells that condition peritumoral zones for subsequent invasion, such as monocyte recruitment via CD40-mediated CCL2 and IFN-γ upregulation or inflammation-driven PSC migration through the CCR10–CCL28 axis (17, 119). Another area deserving of more attention is stage-specific studies in the context of inflammatory-signal-driven immune cell chemotaxis—specifically Th17 and Th2 cells under early PDAC development conditions—or early stage metastatic development, particularly the CCR6–CCL20 axis after initial M2 TAM cultivation in the primary TME and the CCR7–CCL19/21 axis in association with LNMs. Yet an obstacle to this proposal is the current lack of diagnostic efficiency in the healthcare system to adequately detect early-stage PDAC cases and analyze their development in sample sizes large enough to make any significant claims, likely limiting the current scope to murine models. Lastly, there exists a general absence of PDAC-specific models across the different aspects of cancer in the context of CC chemokines, particularly around mapping of different cellular and intracellular sources of CCLs (CCL17, CCL22, CCL20, CCL19, CCL21), MMP expression, and non-nodal metastases, among others. Addressing these shortcomings will bring us closer to understanding the full complexity of PDAC, offer translational insights to other cancer types, and provide a more defined framework for other therapeutic strategies, if not directly identify therapeutic targets themselves.
